# The *Saccharomyces cerevisiae* W303-K6001 cross-platform genome sequence: insights into ancestry and physiology of a laboratory mutt

**DOI:** 10.1098/rsob.120093

**Published:** 2012-08

**Authors:** Markus Ralser, Heiner Kuhl, Meryem Ralser, Martin Werber, Hans Lehrach, Michael Breitenbach, Bernd Timmermann

**Affiliations:** 1 Department of Biochemistry and Cambridge Systems Biology Centre, University of Cambridge, 80 Tennis Court Road, Cambridge CB2 1GA, UK; 2 Department of Vertebrate Genomics and Next Generation Sequencing Core Facility, Max Planck Institute for Molecular Genetics, Ihnestrasse 63-73, 14195 Berlin, Germany; 3 Department of Cell Biology, University of Salzburg, 5020 Salzburg, Austria

**Keywords:** next-generation sequencing, yeast models, phylogeny reconstruction, mapping

## Abstract

*Saccharomyces cerevisiae* strain W303 is a widely used model organism. However, little is known about its genetic origins, as it was created in the 1970s from crossing yeast strains of uncertain genealogy. To obtain insights into its ancestry and physiology, we sequenced the genome of its variant W303-K6001, a yeast model of ageing research. The combination of two next-generation sequencing (NGS) technologies (Illumina and Roche/454 sequencing) yielded an 11.8 Mb genome assembly at an N50 contig length of 262 kb. Although sequencing was substantially more precise and sensitive than whole-genome tiling arrays, both NGS platforms produced a number of false positives. At a 378× average coverage, only 74 per cent of called differences to the S288c reference genome were confirmed by both techniques. The consensus W303-K6001 genome differs in 8133 positions from S288c, predicting altered amino acid sequence in 799 proteins, including factors of ageing and stress resistance. The W303-K6001 (85.4%) genome is virtually identical (less than equal to 0.5 variations per kb) to S288c, and thus originates in the same ancestor. Non-S288c regions distribute unequally over the genome, with chromosome XVI the most (99.6%) and chromosome XI the least (54.5%) S288c-like. Several of these clusters are shared with *Σ*1278B, another widely used S288c-related model, indicating that these strains share a second ancestor. Thus, the W303-K6001 genome pictures details of complex genetic relationships between the model strains that date back to the early days of experimental yeast genetics. Moreover, this study underlines the necessity of combining multiple NGS and genome-assembling techniques for achieving accurate variant calling in genomic studies.

## 2. Introduction

Ageing is common to all living organisms, and knowledge on biochemical and genetic components that accelerate or delay this process are of immense medical interest. Because the lifespan of mammalian organisms is considerably long, short-living species such as the yeast *Saccharomyces cerevisiae* are popular models in experimental ageing research [1,2]. Widely used measures of yeast ageing are (i) chronological lifespan, defined as survival of a stationary culture at 30°C [3], or in its special case 'hibernating lifespan’ at 4°C [4]; and (ii) replicative lifespan (RLS), defined as the number of cell cycles an individual yeast cell can complete [5]. Determination of RLS is time-consuming and technically challenging, as it requires continuous micromanipulation of the target strains [6,7], or single cell trapping and microscopy [8]. To simplify RLS analysis, a genetic assay based on the yeast strain W303-K6001 was introduced around a decade ago, and has become popular [9–14]. The W303-K6001 RLS assay bases on differential expression of the essential *CDC6* gene. Placed under control of two promoters, *CDC6* is always expressed in mother cells (HO promoter), but expressed in daughters only when they grow on galactose (*GAL1* promoter) [11]. Thus, on glucose, daughters arrest, whereas mothers continue to divide until senescence. The cell number in a W303-K6001 glucose microcolony is therefore a direct—and the stationary biomass of a W303-K6001 glucose culture an indirect—measure of RLS [9].

W303-K6001 is a direct descendant of the yeast strain W303-1A, which is commonly used in biomedical research laboratories around the world [15]. This strain is a laboratory mutt that was generated through a series of strain crosses, mainly conducted by Rodney Rothstein during his PhD thesis. W303 derivatives therefore have a complex and not thoroughly documented ancestry. The founding W303 strain W303-1A was derived from W301-18A [16], which was transformed by a plasmid containing the HO gene [17]. W301-18A itself originated from crosses of W87 derivatives [18,19], which are themselves partially descended from yeast strain S288c, the source of the *S. cerevisiae* reference genome [20]. W303-1A further contains genetic material from yeast strains D311-3A [21,22] and a historical yeast strain, D190-9C. Nothing seems to be known about D190-9C, except that it has originated in the laboratory of Jack Szostak (personal communication of R. Rothstein to the *Saccharomyces* genome database, SGD [23]). This complex genealogy mixes with the ancestry of other laboratory strains commonly used today, such as *Σ*1278B and SK1 [24].

Proteomic profiling and tiling microarrays indicated that W303-1A derivatives maintain high similarity to 288c [24,25]. Population genomics confirmed these large genetic similarities, but also revealed the presence of substantial non-S288c material in the W303 genome. For example, on chromosome 2 on the left arm, there is a region similar to west African yeast strains, while a region on the right arm clustered with European strains. Surprisingly, regions that resembled Japanese sake strains were also found in the W303 genome [26]. This genetic divergence probably contributes to physiological differences that have been reported for W303 and S288c derivatives BY4741 and BY4742, the most widespread S288c descendants [27]. These strains differ not only in important physiological parameters such as cell size and volume, but also in their relative plasma-membrane potential and tolerance to alkali-metal cations [28]. Moreover, although S288c strains and W303 have a relatively similar RLS, RLS of W303-K6001 is shortened on glucose media [9,29], and aged W303 cells have considerably larger volumes. As the average protein concentration was reversely changed from 64 pg µm^−3^ (BY4741) to 24 pg µm^−3^ (W303), it was concluded that senescent W303 cells possess larger vacuoles [30].

To provide a comprehensive basis for the interpretation of ageing experiments performed with W303-K6001, and to understand genetic origins and physiological properties of W303-1A derivatives, we constructed a reference genome sequence for W303-K6001. Both high-coverage pysrosequencing (Roche/454) and sequencing by synthesis (Illumina) technologies were then used for highly accurate variant calling against the S288c reference genome. The high sequencing depth (in total 378× average coverage) achieved with both platforms on this 11.8 Mb haploid genome facilitated a profound comparison on their performances for *de novo* assembly and variant calling. Roche/454 sequencing performed better in *de novo* and reference-guided assembly, indicating that the much higher coverage of the short-read technology could not compensate for read length. Both sequencing strategies, however, called a significant number of false positives in variant detection. Merging the outputs of both platforms reduced this number markedly, indicating that, at present, parallel sequencing with more than one NGS technology is essential for generating precise reference genomes and for avoiding false positives in variant detection.

The proportion of the W303-K6001 genome that is highly similar to its main ancestor, S288c, was 85.4 per cent, whereas the remaining genetic material is of different genetic origin. In part, these non-S288c clusters are shared with *Σ*1278B, another commonly used mutt yeast strain. These regions encode for 799 proteins that have altered amino acid sequence compared with S288c.

## 3. Results and discussion

### 3.1. Sequencing and assembly of the W303-K6001 genome

We have previously presented a draft genome sequence for W303-K6001 and one of its variants, K6001-B7. Isolated after chemical mutagenesis, K6001-B7 is a W303-K6001 progeny that exhibits a dominantly inherited increase in resistance to oxidative stress, while having a premature ageing phenotype [13]. This genome sequence was generated by massive parallel pyrosequencing (Roche) and led to the identification of 13 single-nucleotide exchanges that distinguish the parent and its progeny. One of these, a C–T transition in the peroxiredoxin locus *TSA1* (*tsa1-B7*), was shown to be responsible for the stress-resistance phenotype. Here, this W303-K6001 genome draft was improved by including the newest version of the S288c genome (EF4, Ensembl annotated version of S288c genome R64-1-1, GenBank GCA_000146045.2) for reference-based genome assembly. Moreover, we resequenced W303-K6001 using sequencing by synthesis technology (Illumina). We combined the sequencing information obtained with both technologies to correct for platform-dependent sequencing errors (false-positive variant calls) as reported to occur in NGS experiments [31]. To keep experimental variation at a minimum, we used the very same DNA purifications for Roche and Illumina library preparation. At a median read length of 527 bp, we had collected 662.12 Mb of sequence information with the Roche FLX pyrosequencing system. Analysing the W303-K6001 DNA on a Genome Analyzer IIx (GAIIx) with a 120 bp paired-end protocol yielded 3.9 Gb of sequencing data after quality clipping; on average, 101.22 bp per 120 bp GAIIx read were used. The insert size of the paired ends was 225 ± 60 bp (table 1).

**Table 1.** Sequencing of W303-K6001 on two different NGS platforms.

	454 sequencing			Illumina sequencing			combined
	K6001	K6001-B7	total	K6001	K6001-B7	total	
high-quality reads	641 083	684 545	1 325 628	20 407 268	18 266 146	38 673 414	39 999 042
average read length	507.22	492.26	499.48	101.16	101.28	101.22	
median read length	529.0	524.0	527.0				
average insert size (paired end)	single read			225.0	221.3	223.15	
bases (Mb)	325 157	336 965	662 122	2 064 395	1 849 944	3 914 339	4 576 462

Obtained sequencing information was then assembled using the Newbler mapper/assembler v. 2.6, CLC bio reference mapper (included in CLC Genomics Workbench v. 5.1) and SOAPdenovo (63mer v. 1.05). Using Newbler, we tested different strategies for *de novo* assembly of the W303-K6001 genome (table 2). First, we compared the performance of *de novo* assemblies using 454 data alone and in combination of 454 and Illumina data. Interestingly, *de novo* assembly of pure 454 data yielded the largest N50 contig size of 262 kb; adding higher coverage of the Illumina platform did not result in larger contigs. Indicated by a high number of shorter contigs, this might signify that the assembler software was limited in estimating the accurate genome size at this unusual high coverage. Moreover, the insert size distribution of the Illumina paired-end library was below the average read length of the 454 data, and thus did not provide significant additional information for resolving repetitive structures in the yeast genome. Nevertheless, scaffolds produced by the paired-end information of Illumina data resulted in better long-range continuity than the assembly of 454 single reads alone. To exclude that these findings were specific for the Newbler *de novo* assembler, we assembled the Illumina reads also by SOAPdenovo. After optimizing the k-mer value to 57, the results were still lagging behind the Newbler results, however (table 3).

**Table 2.** *De novo* assembly of the W303-K6001 genome.

	*de novo* assembly by SOAPdenovo 63mer	*de novo* assembly by Newbler v. 2.6		combination of reference-guided and *de novo* assembly by Newbler v. 2.6
assembled data	Illumina	454	Illumina + 454	454 + 454 mapped contigs
*contig statistics*				
number of contigs	3095	477	2846	375
number of bases	11 819 873	11 637 892	13 865 032	11 642 694
average contig size	3819	24 398	4871	31 047
N50 contig size	39 717	66 531	42 713	149 943
largest contig size	165 547	260 577	164 236	466 025
*scaffold statistics*				
number of scaffolds	374	—	357	78
number of bases	11 351 824	—	11 856 226	11 591 176
average scaffold size	30 352	—	33 210	148 604
N50 scaffold size	68 612	—	102 849	367 966
largest scaffold size	226 549	—	386 246	833 844

**Table 3.** Reference-guided assembling of the W303-K6001 genome.

	reference-guided assembly by Newbler v. 2.6			reference-guided assembly by CLC reference mapper		
assembled data	Illumina	454	Illumina + 454	Illumina	454	Illumina + 454
*contig statistics*						
number of contigs	268	272	265	593	289	329
number of bases	11 450 524	11 844 486	11 771 493	11 730 567	11 874 714	11 892 270
average contig size	42 725	43 545	44 420	19 782	41 089	36 147
N50 contig size	107 251	261 861	262 228	202 565	231 374	266 295
largest contig size	414 906	666 566	555 078	538 743	743 077	743 360

Next, we assembled the different W303-K6001 datasets by mapping to the *S. cerevisiae* S288c reference sequence (table 3). We obtained higher contig sizes and better long-range continuity than by the different *de novo* assembly approaches, reflecting the high similarity of the two strains. Also, short Illumina reads resulted in lower length coverage of the reference genome than using 454 reads. Additionally, we did apply the CLC bio reference mapper, which gave similar results for 454 and 454 + Illumina reference-guided assemblies, but was better than Newbler when assembling Illumina-only data (table 3).

For the generation of a W303-K6001 reference genome sequence, we combined *de novo* and reference-guided assembly. We did simulate paired-end reads 400 bp in length with insert sizes ranging from 2000 to 4000 bp, based on the reference-guided assembly (Newbler/454 data only) by applying the ‘simulate_reads’ tool (CLC bio). The simulated reads comprised a 10× genome coverage and were *de novo* assembled together with the 454 data. In this way, we did obtain the best ‘*de novo*‘ results, but they were still behind the reference-guided strategy. For this reason, we finally did scaffold the contigs of the reference-guided assemblies according to their positions in the S288c genome. This Whole Genome Shotgun project has been deposited at DDBJ/EMBL/GenBank under the accession ALAV00000000. The functional analysis bases on its first version, ALAV01000000. Moreover, the genome sequence in total, and gene-by-gene-wise, is accessible through the web interface of SGD (http://www.yeastgenome.org [23]).

### 3.2. Combining two next-generation sequencing technologies for SNV calling eliminates a surprisingly high number of platform-specific false positives

To detect variations between S288c and W303 genomes, we performed two independent variant callings using GSMapper 2.6 (Roche). Mapping the pyrosequencing data revealed 11 324 variations, whereas sequencing by synthesis predicted 10 130 differences between the genomes. To eliminate platform-specific errors, both result files were combined. This strategy confirmed solely a number of 9073 differences between the W303-K6001 and the S288c reference genome. These split into 8471 single nucleotide polymorphisms (SNPs), 280 single base insertions/deletions and 322 more complex variants (table 4). Thus, combining both NGS technologies eliminated 3308 variant calls, indicating that 26 per cent of total calls probably represented false positives. Please note that single nucleotide variant (SNV) coordinates refer to the S288c reference genome and must not be used with the reference-based assembly of W303-K6001. For this reason, we provide an additional table with coordinates of SNVs in the W303-K6001 assembly (see the electronic supplementary material, table S1).

**Table 4.** Comparison of S288c and W303-K6001 genomes using two mapping algorithms.

	CLC bio (both technologies)	Newbler (both technologies)	both mappers and both technologies
single nucleotide polymorphism	8815	8471	8049
single insertion/deletion	397	280	25
multiple number variations	370	322	59
sum	9582	9073	8133

We also tested for false-positives created at the stage of mapping. Only 95 per cent (8049) of the SNPs called by Newbler were also called by the CLC bio reference mapper when analysing the same raw data (table 4). On combined Roche and Illumina sequencing data, 766 SNPs called by the CLC biosoftware were not confirmed by Newbler; vice versa, Newbler did not confirm 422 SNPs predicted by the CLC biosoftware. Hence, combination of multiple NGS techniques, but also mapping algorithms, markedly reduces the number of false-positive variant calls.

### 3.3. Comparison of the W303-K6001 genome assembly with whole-genome tiling arrays

**Figure 1.**
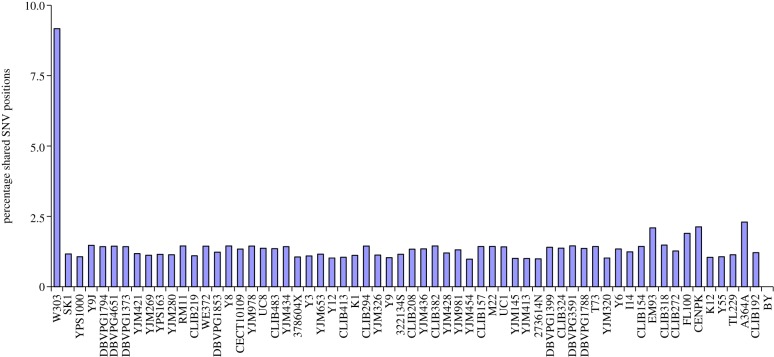
Comparing the W303-K6001 genome sequence with whole-genome tiling arrays. The tiling array correctly identified the yeast background, as a significant number of SNV positions were overlapping. However, the tiling array did not reach sensitivity and accuracy of whole-genome resequencing. All unrelated Non-W303 yeast strains share a number of mutant coordinates, indicating errors in the reference genome or tiling array, or private mutations of the S288c line.

The W303 genome has previously been analysed using whole-genome tiling arrays [32]. To be able to compare our SNP calling with this array, we used Newbler to map the W303-K6001 genome also to the SC genome version EF2 (Ensembl annotated version of R63-1-1, GCA_000146045.1), on which this study was based. Of the 9334 mutant positions, 583 (9.2%) were identified in the tiling array (figure 1). The overlap of the W303-K6001 SNVs with all other analysed yeast strains was around 2 per cent, except for the S288c reference genome. Thus, the tiling array correctly identified the yeast strain and a number of its specific SNV positions. However, a lot of SNVs found by the tiling array study could not be confirmed by our data. In part, this can be explained by the limited accuracy of tiling arrays in detecting the exact position of a SNV in the genome (5 bp window). Interestingly, all analysed yeast strains (except the reference genome strain) on the tiling array share a similar number of variations to the W303-K6001 genome. This number might indicate errors in the EF2 version of the S288c reference genome, or in the design of the tiling array, and thus could represent noise. Consistent with this observation, mapping to EF4 instead of EF2 eliminated 261 SNV calls.

### 3.4. Physiological differences between BY4741 and W303-K6001 explained by its genome sequence

W303-K6001 has a short RLS [9]. This property might have an impact on ageing studies in general, but facilitates a quick assay to determine RLS phenotypes by simply counting the cell numbers in glucose microcolonies. The Newbler variant calling identified several mutations within ageing factors, identifying proteins that might contribute to this phenotype. Compared with S288c, 799 K6001-W303 proteins have altered sequence. In 432 proteins, only one or two residues differ. Largely, these might represent natural allelic variations that cause only minor physiological effects. However, in 239 proteins, three or more amino acids are exchanged (see the electronic supplementary material, table S2), and the list of mutations continues with 41 small insertions/deletions, and 87 more complex variations. Several of the latter may lead to a loss of the protein function as they affect the reading frame. Identified mutant genes include *ade2-1, trp1-1, can1-100, leu2-3,112* and *his3-11,15* alleles, which were actively crossed into W303-1A as auxotrophic markers. The genome sequencing identified either nonsense mutations (*ade2* and *trp1*) or frameshift mutations (*can1*, *leu2*, *his3*) as cause of their loss of function (table 5). In contrast to W303, BY4741/BY4742 are wild-type for *TRP1* and *ADE2*, but deficient in *met15* or *lys2*, respectively. As these mutations all block central and essential metabolic pathways [33], it is likely that they make a major contribution to the physiological differences reported for W303 and BY4741 [28,30].

**Table 5.** Auxotrophic marker mutations found in W303-K6001.

allele name	locus	detected mutation
*ade2-1*	YOR128C	nonsense, *glu64STOP*
*trp1-1*	YDR007W	nonsense, *glu83STOP*
*can1-100*	YEL063C	frameshift, *lys47*
*leu2-3,112*	YCL018W	frameshift, *gly83*
*his3-11,15*	YOR202W	2x frameshift, *ala70* and *glu106*

The list of genes containing frameshift mutations also includes genetic factors that have been implicated in ageing-related physiological processes (table 6 lists genes belonging to gene ontology categories with more than two genes carrying a frameshift mutation). We detected four non-synonymous mutations, one nonsense mutation and two frameshift mutations within the coding sequence of the *MET1* gene involved in methionine biosynthesis. This pathway is used for auxotrophic selection, and closely connected to the oxidative stress defence, glutathione as well as homocysteine metabolism [34]. The nonsense mutation terminates Met1p at its penultimate amino acid, and the two frameshifts are in close proximity so that the second one restores the open reading frame. Indeed, *met1*-*W303* appears to be at least partially functional, as the strain is methionine prototroph (data not shown).

**Table 6.** Gene ontology (GO) categories containing two or more genes with a single nucleotide insertion or deletion.

GO ID	GO term	frequency	genome frequency	gene(s)
2181	cytoplasmic translation	five of 41 genes, 12.2%	174 of 6311 genes, 2.8%	*RPL28,RPL34B,RPL13B,RPS16A,RPS19B*
42274	ribosomal small subunit biogenesis	three of 41 genes, 7.3%	124 of 6311 genes, 2%	*NOP19,RPS16A,RPS19B*
6520	cellular amino acid metabolic process	three of 41 genes, 7.3%	240 of 6311 genes, 3.8%	*LEU2,MET1,HIS3*
6811	ion transport	two of 41 genes, 4.9%	132 of 6311 genes, 2.1%	*MTM1,YHL008C*
6364	rRNA processing	two of 41 genes, 4.9%	294 of 6311 genes, 4.7%	*NOP19,RPS16A*
32200	telomere organization	two of 41 genes, 4.9%	67 of 6311 genes, 1.1%	*TEL2,EST1*

In this context, we noticed that the phenomena of co-occurring frameshift mutations that neutralize each other was not unique among gene ontology terms related to ageing (table 6). We found two neutralizing frameshifts within the telomere organizers *TEL2* (12 SNPs and two neutralizing frameshifts), *EST1* (two neutralizing frameshifts) and the manganese carrier *MTM1* (two neutralizing frameshifts). Telomere organization has been closely associated with ageing in vertebrates, and in yeast their length is kept constant during replicative ageing [35]*. MTM1* is required for the mitochondrial activation of superoxide dismutase (*SOD2*) and oxidative stress resistance [36]. Thus, this complex mutation appears to have occurred more than once in the history of W303. It is likely that the altered amino acids sequence within the frameshifts has influence on the functionality of these genes.

Other genes related to ageing that contain frameshift mutations include eight proteins involved in translation and the biogenesis of the small ribosomal subunit, and YHL008C, a protein that is involved in chloride ion uptake [37,38]. Thus, although the artificial expression of *CDC6* might explain large parts of the W303-K6001 lifespan, the strain carries several other mutations within genes involved in this biological property.

### 3.5. Phylogeny of W303-K6001

**Figure 2.**
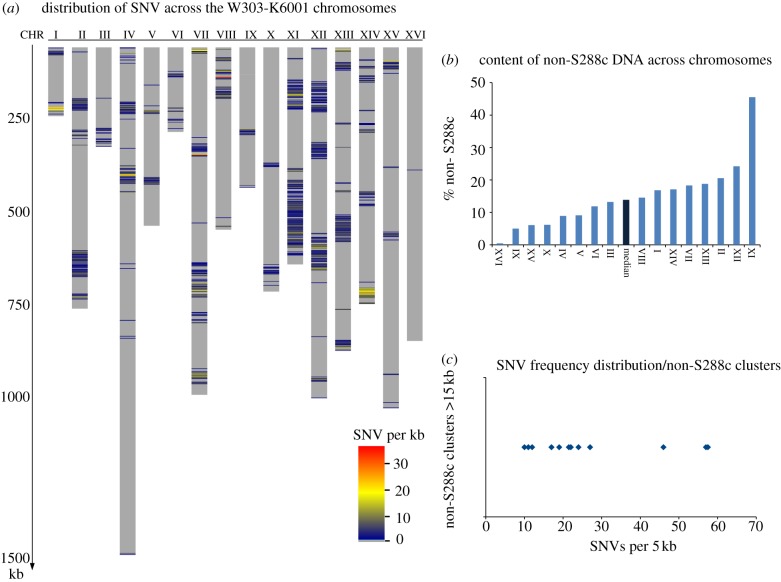
Unequal SNV distribution in the W303 genome illustrates its mutt ancestry. (*a*) Regions with high-sequence divergence to S288c cluster together. Chromosomal sequences with high identity (less than or equal to 0.5 SNVs per kb) to the S288c Reference genome EF4 are depicted in grey, indicating that 85.4% of the W303-K6001 genome is a S288c descendant. Regions with higher variability form clusters. (*b*) Median percentage of genetic material with greater than 0.5 SNV per kb divergence from S288c, per chromosome. (*c*) Distribution of SNV frequencies per 5 kb segment, taking into account all non-S288c clusters larger than 15 kb.

As mentioned earlier, W303 is a mutt of different laboratory strains, including S288c/W87, D311-3A and D190-9C [18,19,21,22]. Proteomic profiling, tiling arrays and what is known about its history indicated that most of the W303 background is S288c-like [24,25]. The W303-K6001 genome sequence allowed us to define the regions that derived from S288c, as they are virtually identical to the reference genome (less then 0.5 SNV per kb; figure 2*a*). In contrast, the sequence reveals distinct clusters of much higher genetic variability, identifying the genetic material derived from other parents. In total, the clusters with sequence divergence larger than 1 SNV per kb span 1744 kb, corresponding to 14.6 per cent of the W303-K6001 genome. The differences between the chromosomes are, however, relatively large. W303-K6001 chromosome XVI, for instance, is virtually identical to S288c, whereas just half of chromosome XI is S288c-like (figure 2*b*). Chromosome XVI is also an indicator of the genome stability of W303-K6001; except for a small cluster that spans 0.4 per cent of its sequence, there is virtually no variation compared with the S288c reference genome. Thus, the approximately four decades since the divergence of W303 and S288c did not lead to a significant number of secondary genetic changes, indicating that W303-K6001 still resembles the status of W303-1A after the crosses that led to its generation [15]. However, this also implies that smaller molecular incompatibilities (i.e. those that might be caused by non co-evolved subunits of protein complexes) might still exist in the W303 genome and impact its robustness.

**Figure 3.**
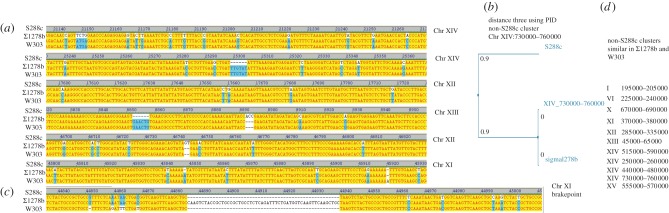
W303-K6001 contains clusters that are identical to *Σ*1278B, but differ in S288c. (*a*) S288c is the main ancestor parent of W303 and *Σ*1278B; however, part of the non-S288c-derived W303-K6001 genome is also found in *Σ*1278B. Shown are two exemplary multiple alignments each from Chr. XIV, XIII and XI, and the 3′ breakpoint of the cluster on Chr XI. (*b*) Distance diagram of S288c, *Σ*1278B and W303-K6001 for the non-S288c cluster on Chr XIV 730 000–760 000. (*c*) W303-K6001: non-S288c sequence clusters with high sequence similarity to *Σ*1278B.

The median percentage of non-S288c-like DNA sequences per W303 chromosome was 13.9 per cent (figure 2*b*). Analysing the SNP frequency in clusters greater than 15 000 bp, we noticed that most have a median difference of two to five variations per kb (no chromosomal region had a difference between 0.5 and 2 SNVs per kb), but three clusters had higher median divergence of 9–12 SNVs per kb (figure 2*c*). One could speculate that for this reason one might be able to assign the origin of these regions to a different ancestor; however, the low number of these highly divergent clusters might equally point to regions that were exposed to different selection pressure. We then performed a number of BLAST searches, using the blastall v. 2.2.24 tool [39], querying the yeast genome resources available at SGD January 2012 [23]. Confirming the results from Liti *et al.* [26], several non-S288c clusters had similarities to quite different yeast genomes, including sake strains. Interestingly, we found that a large non-S288c cluster on chromosome XIV (730 000–760 000) was not only similar, but identical, to the genome sequence of another yeast strain commonly used in research, *Σ*1278B [40]. Exemplary ClustalW-generated alignments (figure 3*a*) and a corresponding distance diagram (figure 3*b*) are illustrated. Extending our search, we could then identify several other regions in the W303 genome that were similar to the *Σ*1278B genome (figure 3*c*). We illustrate one exemplary breakpoint of the Chr XI cluster, where the W303 genome switches from a non-S288c and non-*Σ*1278B region to a *Σ*1278B-like genome, indicating differing phylogenetic origins of this cluster (figure 3*d*). As *Σ*1278B shares part of its genealogy with S288c (47% of its genome does not differ from S288c [24]), these results indicate that W303 and *Σ*1278B share a second ancestor, or (less likely) that a third strain contributed to the genome of S288c after W303/*Σ*1278B split from the lineage.

## 4. Concluding remarks

The K6001-W303 genome sequence facilitates comprehensive insights into its physiology and genealogy. In comparing W303-K6001 and S288c genome sequences, cross-platform sequencing and mapping eliminated 3308 false-positive nucleotide calls and 2389 mapping artefacts. The resulting consensus genome sequence differs in 8133 positions (including 8049 SNPs) from the S288c genome. These data demonstrate that it is vital to reproduce genetic variations identified by different sequencing strategies. Generated by crossing mutt yeast strains W87, D311-3A and D190-9C, W303-K6001 remains closely related to S288c, sharing 85.4 per cent of its genome. Remaining genetic differences cause altered amino acid composition or reading frame in 799 proteins, some of high relevance for physiology and ageing. Individual studies now have to clarify to what extent these mutations contribute to the physiological differences between these common yeast backgrounds. Unequal genomic SNV distribution allowed conclusions on the W303 genealogy, and identified a close genetic relationship of W303 with *Σ*1278B. This strain also shares 47 per cent of its background with S288c, but overall differs from it in 3.2 SNPs per kb, which cause 44 genes to be uniquely essential to *Σ*1278b and 13 to S288c [40]. Because W303-K6001 and *Σ*1278B share genomic regions not found in their common ancestor, it is likely that they have a second mutual ancestor. Thus, W303 represents a partial hybrid of S288c and *Σ*1278b, shedding new light into the complex relationships of today's widely used laboratory strains.

## 5. Material and methods

The 454-genome draft sequence of W303-K6001 [11] (*MAT*a; *ade2-1, trp1-1, can1-100, leu2-3,112, his3-11,15, GAL, psi+, ho::HO::CDC6 (at HO), cdc6::hisG, ura3::URA3 GAL-ubiR-CDC6 (at URA3*)) and K6001-B7 [13] (*MAT*a; *ade2-1, trp1-1, can1-100, leu2-3,112, his3-11,15, GAL, psi+, ho::HO::CDC6 (at HO), cdc6::hisG, ura3::URA3 GAL-ubiR-CDC6 (at URA3) tsa1-B7*) was published earlier [13].

### 5.1. Illumina sequencing

Genomic DNA from both strains was sheared by sonification to fragment sizes of around 225 bp, cleaned (Zymo Research) and universal sequencing adaptors were ligated. After library quantification at a Qubit (Invitrogen), a 10 nmol stock solution of the amplified library was created. We loaded 8 pM of the stock solution onto the channels of a 1.4 mm flow cell, and cluster amplification was performed. Sequencing-by-synthesis was performed on an Illumina Genome Analyzer (GAIIx). After quality control of the first base incorporation (signal intensities, cluster density), the run was started. All samples were subjected to 120 bp paired-end sequencing.

### 5.2. Data analysis

#### 5.2.1. Raw data processing of 454 reads

After default raw data processing, we used a resequencing trimming filter to increase the data output. (parameters: doValleyFilterTrimBack = false, vfBadFlowThreshold = 6, vfLastFlowToTest = 168, errorQscoreWindowTrim = 0.01). With these parameters, we got an average quality score of greater than Q30 per base.

#### 5.2.2. Raw data processing of Illumina GAIIx sequence reads

Illumina data were provided as qseq files generated by the Bustard 1.8.0 pipeline. High-quality data were extracted using homemade scripts (perl/awk). As a first step, reads were trimmed in such a way that only the longest sequence range of the reads, which did not contain bases of quality lower than Phred 12, was used. Additionally, adaptor sequences were clipped, if at least 15 bp of the adaptor's 3′ end was found in each read. After trimming and adaptor removal, only reads equal to or longer than 64 bp were used in the mapping/*de novo* assemblies. In a final step, most of the duplicate reads resulting from amplification bias during library construction were removed, if the first 64 bases of the reads were identical. Finally, sequence data were stored in fasta files with Newbler-compatible headers.

#### 5.2.3. Assembly and mapping

Assemblies were computed by the Roche/454 Newbler v. 2.6 assembler or mapper software applying default parameters. Additional *de novo* assemblies were performed by the 127mer or 63mer version of SOAPdenovo (v. 1.05, downloaded from http://soap.genomics.org.cn). After running several assemblies, we found that a kmer size of 57 was giving the best results in terms of N50 contig sizes after scaffolding and gap filling and in terms of total consensus length. Additional reference-guided assemblies were carried out by the CLC Genomics Workbench v. 5.1 (CLC BIO, Aarhus, Denmark), and its reference mapper and probabilistic variant detection modules (default parameters for the mapping algorithm, for variant detection the ploidy parameter was set to 1).

## 6. Acknowledgements

We thank Steve Oliver (University of Cambridge) and our laboratory members for support and critical discussions. We acknowledge funding from the Max Planck Society, the Wellcome Trust (no. RG 093735/Z/10/Z) and the ERC (Starting grant 260809). Markus Ralser is a Wellcome Trust Research Career Development and Wellcome-Beit prize fellow.

## Supplementary Material

SNP cooridnates of W303-K6001 and S288c

## Supplementary Material

Mutations per gene, table

## Supplementary Material

Genome Sequence of W303-K6001
